# Renal perfusion pressure: role and implications in critical illness

**DOI:** 10.1186/s13613-025-01535-y

**Published:** 2025-08-08

**Authors:** Rakshit Panwar, Bairbre McNicholas, J. Pedro Teixeira, Amit Kansal

**Affiliations:** 1 Intensive Care Unit, John Hunter Hospital, Newcastle, Australia; 2 School of Medicine and Public Health, University of Newcastle, Newcastle, Australia; 3 Intensive Care Unit, Galway University Hospital, Galway, Ireland; 4 University of Galway, Galway, Ireland; 5 Division of Nephrology, Department of Internal Medicine, University of New Mexico, Albuquerque, NM USA; 6 Center for Adult Critical Care, University of New Mexico Hospital, Albuquerque, NM USA; 7 Department of Intensive Care Medicine, Ng Teng Fong General Hospital, National University Health System, Singapore, Singapore; 8 Department of Biomedical Informatics, National University of Singapore, Singapore, Singapore

**Keywords:** Renal perfusion pressure, Targets, Renal blood flow, acute kidney injury, Septic shock, Vasopressor therapy, Renal autoregulation, Renal microcirculation

## Abstract

The pressure-flow relationship is fundamental to circulatory hemodynamics of any organ. In the kidney, renal perfusion pressure (RPP), defined as the gradient between mean arterial pressure and renal venous pressure or mean systemic filling pressure, serves as the principal driving pressure for renal blood flow (RBF). This concept recognizes that both arterial hypotension and venous congestion can reduce the pressure gradient for renal perfusion, potentially contributing to renal dysfunction or acute kidney injury (AKI). In health, whenever RPP fluctuates, the kidney autoregulates intrarenal vascular resistance to maintain stable RBF and glomerular filtration rate over a range of RPP. However, in critical illness, autoregulatory capacity may be impaired, and the degree of impairment can vary not only between patients but also within the same patient depending on the disease context or stage of illness. Therefore, during critical illness, inadequate RPP tends to overwhelm renal autoregulation capacity earlier than anticipated, leading to tissue hypoperfusion and increased risk of AKI. Relying on standard blood pressure targets to optimize RPP may not account for such inter- or intra-individual variations in autoregulation. Experimental models have shown that AKI can develop without overt macrocirculatory changes, implicating microcirculatory dysfunction as an important contributor too. Dynamic, multi-modal assessment of renal perfusion may offer a more precise approach to renal protection. Additionally, the focus of research has shifted towards providing new insights into individualized perfusion targets and refining RPP-guided strategies to prevent AKI among high-risk patients in ICU. The objective of this review is to describe the role of RPP, implications of dysregulated renal perfusion, approaches to monitoring renal perfusion, and potential therapies targeting RPP on the horizon for critically ill patients.

## Renovascular anatomy

**Fig. 1:**
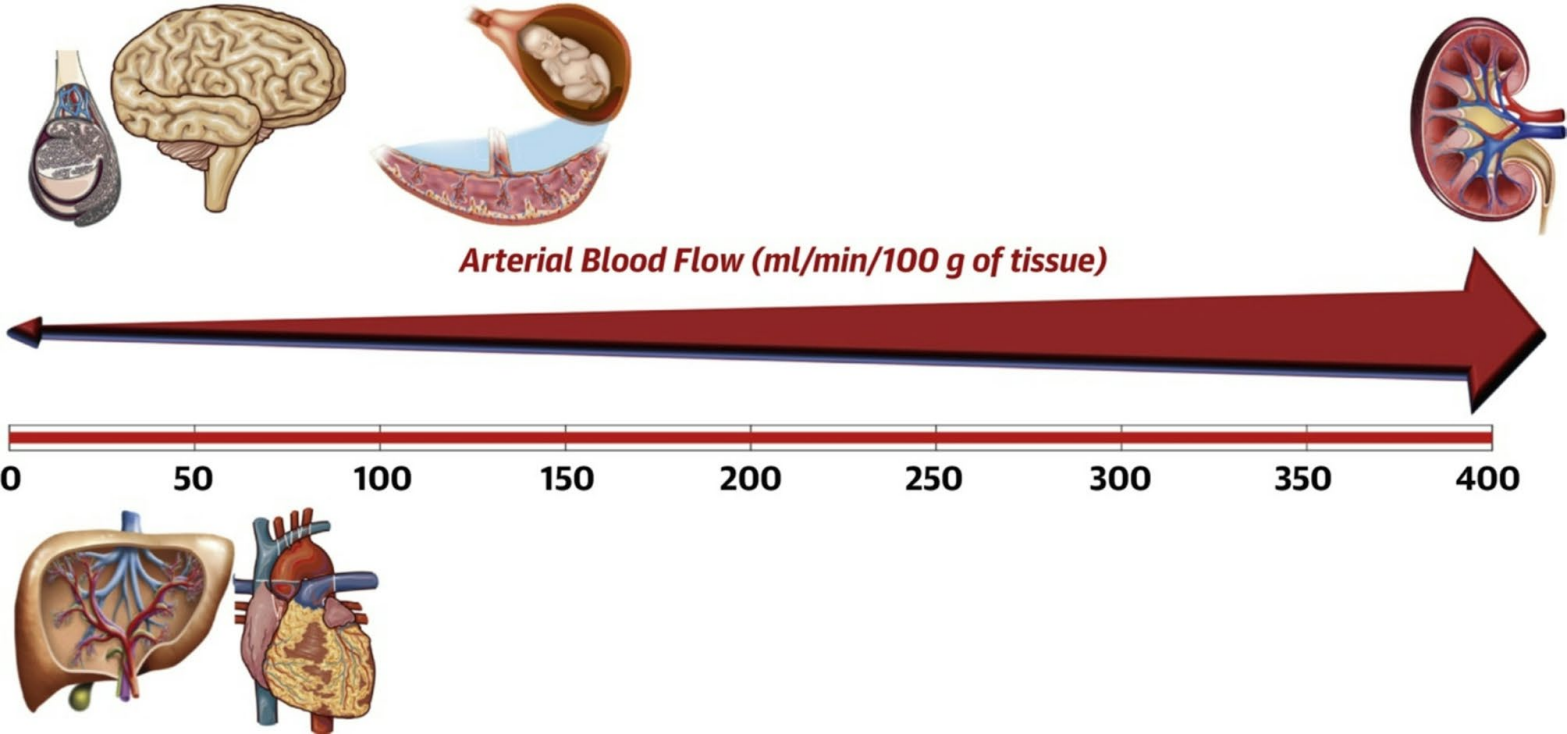
Renal blood flow relative to other vital organs Note: This figure illustrates the relative perfusion of key organs, highlighting the kidneys as having the highest arterial blood flow per unit tissue mass. Reprinted from the *Journal of the American College of Cardiology* [220], Vol. 74, Issue 9, Julio A. Chirinos, Patrick Segers, Timothy Hughes, Raymond Townsend, Large-Artery Stiffness in Health and Disease: *JACC State-of-the-Art Review*, pp. 1237–1263, Copyright ©2019, *with permission from Elsevier*

The kidneys receive approximately 20–25% of the cardiac output - roughly 1 L per minute of blood flow (Fig. 1). Despite such high blood flow, overall oxygen extraction is relatively low (10% of oxygen delivery (DO_2_)) compared to the heart or brain, because the vast majority of the high blood flow is used for filtration rather than metabolic activity [1]. The renal arterial system comprises macrocirculatory components, including the renal arteries and their branches, as well as the renal microcirculation, which includes two primary capillary networks, the glomerular capillaries and the peritubular capillaries, along with their associated arterioles [2, 3]. 

The nephrovascular unit consists of nephrons and their associated vasculature (glomerular and peritubular capillaries), integrating blood flow with tubular function. The arterial network supplying the nephrons resembles a rooted tree, branching hierarchically from the renal artery to the afferent arterioles, ensuring precise regulation of glomerular filtration and tubular reabsorption [4]. Afferent arterioles feed the glomerular capillaries, which are drained by efferent arterioles. These efferent arterioles give rise to peritubular capillaries surrounding the tubules, which, in the medulla, form the vasa recta. Glomerular capillaries mediate filtration, peritubular capillaries facilitate tubular reabsorption and secretion, and the vasa recta enable countercurrent exchange and arteriovenous oxygen shunting [5, 6]. The vasa recta also help establish the corticomedullary osmotic gradient essential for the urinary concentrating ability of the kidneys [7]. 

Glomeruli feed plasma filtrate into nephrons - the functional units of the kidney. There are approximately 1 million nephrons per kidney in humans; this number is reduced in hypertensive patients [8, 9]. The cortex of the kidney receives the majority of blood flow and has higher tissue oxygen concentration, whilst the outer medulla receives about half that flow and has a lower tissue oxygen concentration, making it vulnerable to hypoxia due to countercurrent exchange or arteriovenous shunting [6]. These structural features have important clinical implications, as they contribute to regional differences in susceptibility to ischemia, irreversible loss of nephrons following vascular occlusion, and heightened sensitivity of the medulla to hypoxic or ischemic injury [4]. 

## Renal perfusion pressure and physiology of autoregulation

Renal perfusion pressure (RPP) is the effective pressure gradient driving renal blood flow (RBF), and is typically estimated as the difference between mean arterial pressure (MAP) and either intrarenal venous pressure or mean systemic filling pressure (MSFP) or central venous pressure (CVP) as a surrogate [10]. CVP reflects pressure in the thoracic vena cava near the right atrium and is influenced by multiple factors, including intrathoracic pressure, right heart function, and volume status. MSFP, in contrast, is a theoretical construct representing the pressure in the systemic circulation when there is no flow, reflecting the elastic recoil of the vasculature and stressed blood volume - effectively, the driving pressure for venous return [11]. While MSFP offers a more physiologically grounded basis for estimating the downstream component of RPP, it is difficult to measure directly in clinical settings. Surrogate equations (e.g. MSFP = 0.96 x CVP + 0.04 x MAP + c x CO) have been proposed [12], however, this model assumes fixed weightings for the contribution of CVP and MAP, which may not reflect inter-individual variability in vascular tone or compliance, particularly in critical illness. Moreover, the fact that CVP itself is heavily weighted in this estimate reintroduces its limitations into the MSFP calculation. Estimation of MSFP using inspiratory hold-based methods or extrapolation from venous return curves remain largely experimental and have not been brought into routine clinical practice [13]. CVP is used as a surrogate for renal venous pressure, but it is likely that MAP- MSFP is a better representation, although technically challenging, of the true perfusion gradient across the kidney [10]. 

**Fig. 2:**
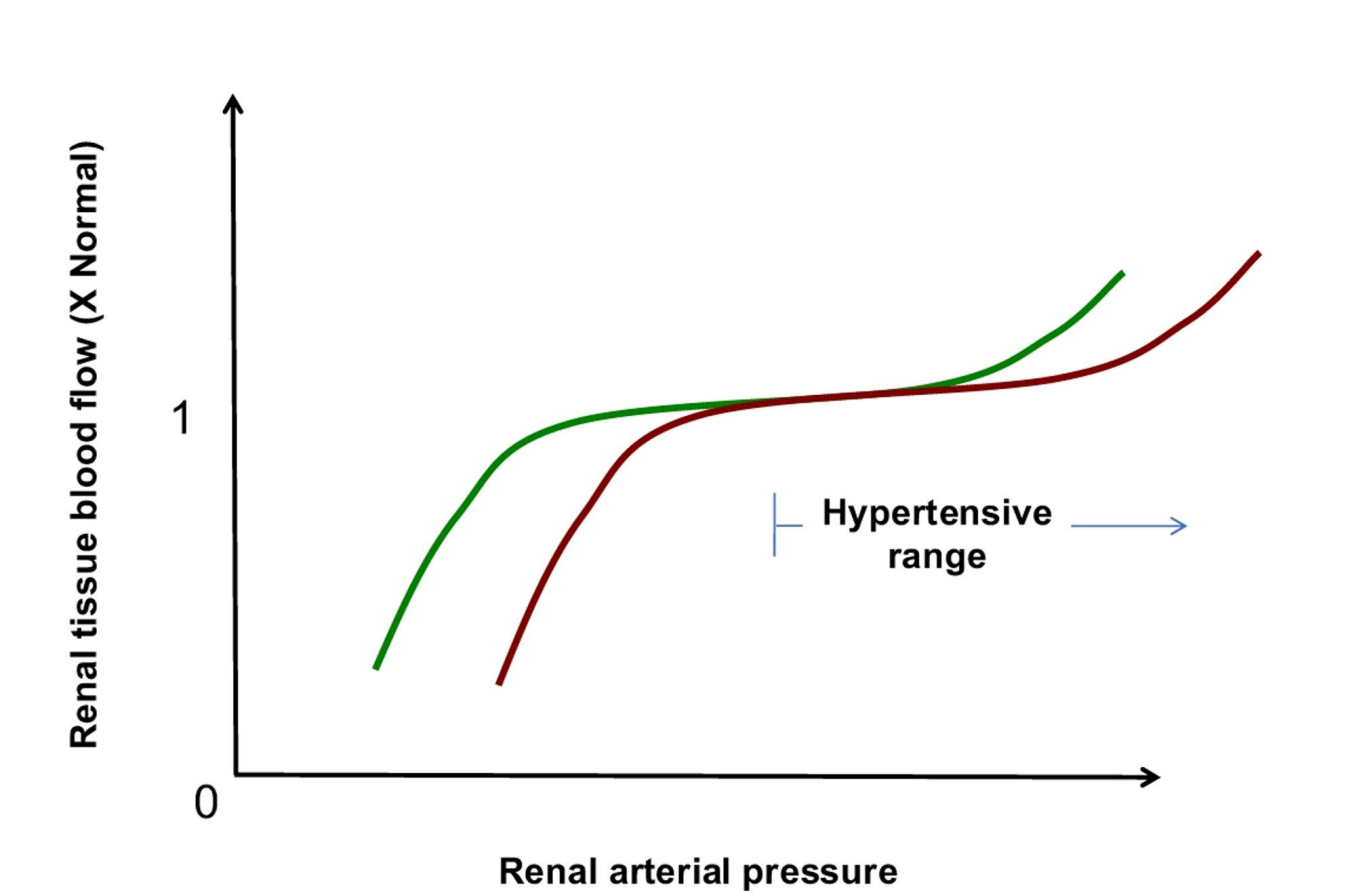
Autoregulation in usual health Note: This schematic diagram illustrates the pressure-flow relationship of renal blood flow (RBF) with renal arterial pressure under normal conditions. Under physiological conditions, tissues autoregulate their blood flow to align with metabolic demands, maintaining relatively constant perfusion despite fluctuations in perfusion pressure - approximately within a range of 60 to 150 mm Hg. When pressure drops below the lower limit of autoregulatory threshold, flow becomes pressure-dependent rather than demand-driven, heightening the risk of ischemia in metabolically active regions. In chronic hypertension, however, adaptive changes such as microvascular remodeling, capillary rarefaction, and increased vascular resistance shift the autoregulatory curve rightward. As a result, the lower limit of effective autoregulation rises, making 60 mm Hg an inadequate perfusion pressure for the kidneys and other vital organs in these patients. While this shift is well recognized in theory, its integration into routine clinical decision-making remains inconsistent

Factors influencing RPP include systemic arterial pressure, renal vascular resistance, venous congestion, neurohumoral regulation, and local metabolic signals. Normal renal function requires tubular flow to remain within a narrow physiological range, and deviation from this range can compromise nephron function. The kidney maintains RBF and glomerular filtration rate (GFR) across a wide range of arterial pressures through autoregulation (Fig. 2) - primarily via an enhanced myogenic response in renal arterioles, contributing ~ 50% of autoregulation, and tubuloglomerular feedback (TGF), which accounts for ~ 35%, with other less well understood mechanisms accounting for the rest [14–17]. 

All vascular beds exhibit some degree of autoregulatory response to changes in perfusion, usually mediated by a myogenic mechanism, in which the mechanical forces exerted upon the walls of an arteriole in the setting of increased perfusion trigger contraction of vascular smooth muscle cells [16]. The myogenic response involves rapid adjustments in afferent arteriole tone - vasoconstriction or dilation - within seconds of local pressure changes [14]. TGF, a kidney-specific negative feedback system, operates on a slower timescale of approximately 30 s. It regulates single-nephron GFR based on chloride concentrations sensed at the macula densa via the apical Na-K-2Cl (NKCC2) cotransporter, the pharmacologic target of furosemide and other loop diuretics [18]. The macula densa detects changes in sodium delivery through chloride sensing channel and then modulates afferent and efferent arteriolar tone to stabilise GFR. This feedback loop also effects renin release and adjusts arteriolar tone through paracrine mediators such as adenosine, nitric oxide, and prostaglandins [18, 19], tying tubular flow to vascular resistance [20] and ultimately restoring RBF back towards its physiological set point [19]. 

In addition to these autoregulatory mechanisms that are largely intrinsic to the kidney, RBF is highly regulated by a variety of additional autocrine, paracrine, and hormonal factors. These mediators can be broadly categorized as vasodilatory or vasoconstrictive. Vasodilatory substances include nitric oxide (NO) and locally-produced prostaglandins (e.g., prostaglandin E_2_ and prostacyclin) that play a role in maintaining RBF in the setting of hypoperfusion states [21, 22]. Vasoconstrictive factors include renal sympathetic nerve activity as well as circulating norepinephrine, angiotensin II, endothelins, vasopressin, and vasoconstrictive prostaglandins (e.g., thromboxane) [21, 22]. 

Importantly when considering the impact of these vasoactive substances on RBF and GFR, the primary site of activity - afferent versus efferent arteriole - is vitally important. Vasoconstrictors that act primarily upon the afferent arteriole (e.g., sympathetic nerves/ norepinephrine or endothelin) will tend to reduce both RBF and GFR, whereas vasodilators that act primarily upon the afferent arteriole (e.g., nitric oxide and vasodilatory prostaglandins) will tend to increase both parameters [23]. In contrast, vasoactive agents that act primarily upon the efferent arteriole will have opposing effects on RBF and GFR. For example, vasoconstrictors such as angiotensin II and vasopressin will tend to decrease RBF but, as they constrict outflow from the glomerular capillaries, tend to maintain glomerular pressure and GFR [23, 24]. 

However, while these local effects influence how these vasoactive agents tend to modulate RBF and GFR, the overall effect of exogenous vasoconstrictors or vasodilators will also depend on their systemic effects. For example, though norepinephrine tends to reduce RBF when infused to subjects with normal hemodynamic status or hypovolemia, it tends to maintain or even increase RBF when given in hyperdynamic septic shock by correcting pathologic vasodilation and/or restoring RPP, thereby potentially improving kidney function [25–31]. Conversely, the direct effect of angiotensin-converting enzyme inhibitors or angiotensin receptor blockers would tend to increase RBF, but if administered to patients with pre-existing hypotension or hypovolemia the resulting decrease in RPP could reduce RBF.

## Pathophysiology of renal hypoperfusion

In the early stages of reduced RBF with corresponding drop in GFR, the kidney enters a state of ‘self-preservation’ characterized by permissive azotemia [32]. This adaptive response may be protective: it decreases the delivery of inflammatory mediators into the tubules [33] and reduces the tubular solute load and subsequent reabsorption, thereby lowering metabolic strain by reducing tubular oxygen demand [34]. 

**Fig. 3:**
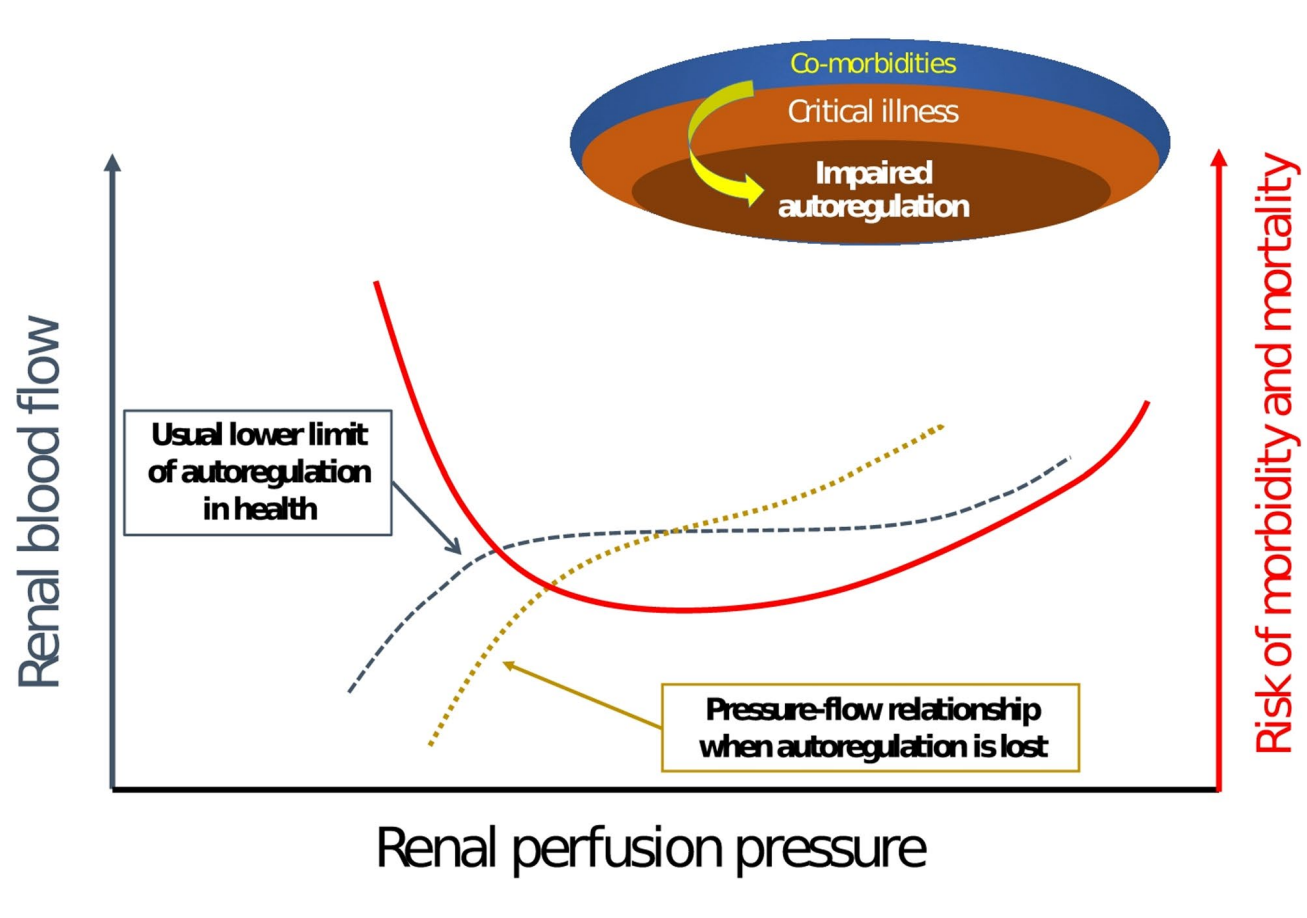
Vulnerability of renal autoregulatory capacity during critical illness Note: This schematic diagram illustrates the pressure-flow relationship in states of impaired autoregulation such as critical illness or in patients with comorbidities. In health, intact autoregulation ensures renal blood flow (RBF) remains constant despite fluctuations in renal perfusion pressure (RPP). However, in critical illness, autoregulation may be impaired or lost, resulting in a direct pressure-flow dependency as shown. Consequently, RBF may be significantly reduced at RPP values that are conventionally considered adequate. Autoregulatory capacity, when compromised, is associated with increased morbidity and mortality (bold red line). This illustrates the potential inadequacy of a fixed RPP threshold and the importance of individualized targets.

If the pathological insult persists, hypoperfusion ensues once RPP falls below the lower autoregulatory threshold - or even within a normal range if autoregulation is impaired as commonly seen in critical illness (Fig. 3) - resulting in reduced DO₂ and renal tubular ischemia [35]. Likewise, inadequate transglomerular pressure gradient, due to increased afferent arteriolar resistance, efferent arteriolar dilation, or both, further reduces GFR. These changes collectively impair adenosine triphosphate delivery to metabolically active tubular epithelial cells, exacerbating tubular injury [36]. 

Additionally, reduced RBF also invokes a mechanism of increasing the filtration fraction (FF) to maintain GFR. The FF represents the amount of RBF being filtered in the Bowman’s space (FF = GFR/RBF) [37]. A higher FF increases proximal tubular sodium and water reabsorption, as tubular transport is highly load-dependent [38]. There is a close linear correlation between GFR, sodium reabsorption, and oxygen consumption (VO₂), implying that increased tubular workload for reabsorption requires higher tubular O_2_ consumption [39]. Notably, at baseline, 70–80% of renal VO₂ supports active sodium reabsorption [40]. In states of hypoperfusion, this demand-supply mismatch aggravates tubular damage.

### Macro- and micro-circulation

Large epidemiological studies report that 25–88% of patients with septic AKI require vasoactive support at the time of diagnosis, suggesting that macrocirculatory abnormalities are common in patients with human septic AKI [41–44]. Phase-contrast MRI studies performed within 48 h of ICU admission have demonstrated reduced renal blood flow in septic AKI, indicating that macrocirculatory compromise may occur early [45]. However, some experimental models have shown that AKI can develop without overt macrocirculatory changes, implicating microcirculatory dysfunction as an important contributor too [46]. Notably, these animal studies often avoided or mitigated hypotension through early fluid or vasopressor therapy. In contrast, in clinical practice, sepsis is often not recognised until after hemodynamic compromise or organ dysfunction is evident. Several human and animal studies have demonstrated that interventions aimed at improving macrocirculatory parameters, particularly vasopressors, may favourably influence the renal microcirculation [25, 47–49]. Therefore, a reasonable interpretation is that septic AKI arises from a complex interplay between macro- and micro-circulatory factors. While sepsis accounts for a substantial proportion of AKI in the ICU, other causes such as surgery, shock states, drug toxicity, or hepatorenal or cardiorenal syndrome [50–52] are also common, and the role of microcirculatory dysfunction in these settings remains less well characterised [53]. 

### Renal interstitial pressure

An increase in renal venous pressure will be conducted backwards and lead to renal parenchymal congestion within the non-distensible renal capsule. Renal interstitial pressure will also be elevated with tubular injury, which causes significant filtrate leakage and intra-renal inflammation due to disruption of tubular tight junctions and tubular obstruction [54, 55]. Studies suggest the role of lipopolysaccharide-induced disruption of the tight junctions in proximal tubules, resulting in filtrate leakage from the proximal tubular lumen into the interstitium in septic AKI [56]. Tubular injury also diminishes reabsorptive efficiency and contributes to luminal obstruction, further elevating interstitial pressure and impairing renal function. These processes create a vicious cycle: tight junction disruption leads to more filtrate escape, amplifying interstitial pressure and worsening tissue injury.

### Intra-abdominal hypertension

Extra-renal stress due to increased intra-abdominal pressure (IAP) leads to decreased RBF, GFR, tubular function, and urine output [57]. Normal IAP is approximately 5–7 mmHg, whereas intra-abdominal hypertension is defined as sustained IAP of ≥ 12 mmHg, with abdominal compartment syndrome defined as intra-abdominal hypertension leading to new organ dysfunction (usually with IAP > 20 mmHg) [57, 58]. As IAP rises above central venous pressure (CVP) and renal venous pressure, it is transmitted to renal veins, effectively becoming the downstream pressure for renal perfusion (i.e., RPP = MAP– IAP) [59]. In fact, the kidneys are so susceptible to increased IAP that abdominal compartment syndrome is considered unlikely in the absence of oliguria [51]. While abdominal compartment syndrome is characteristically described in patients with acute abdominal pathology or after major abdominal surgery, recent data suggest that intra-abdominal hypertension plays a role in the pathogenesis of hepatorenal syndrome and AKI in general in patients with liver disease. Multiple studies have found high rates of intra-abdominal hypertension in patients with cirrhosis, especially among the critically ill [60–62]. 

### Urinary tract obstruction

Urinary obstruction initially triggers a compensatory increase in RBF, likely mediated by kidney-derived prostaglandin E_2_, to preserve GFR. This response is more pronounced in bilateral than in unilateral obstruction [63]. However, within hours, RBF begins to decline while intratubular pressure continues to rise. As RBF progressively diminishes, GFR falls, and intrarenal blood flow redistributes from the cortex to the medulla. Sustained obstruction under these conditions leads to acute tubular injury and, if unrelieved, can result in irreversible renal damage [36]. 

### Impact of renal replacement therapy

While lifesaving, renal replacement therapy (RRT) may itself contribute to AKI [64]. It can reduce GFR and cause tubular stress, potentially leading to worsening oliguria and delayed renal recovery [65]. These effects appear to be mediated by hypoperfusion, particularly due to excessive ultrafiltration - either from higher intensity of RRT or faster net ultrafiltration rates [66, 67]. Additionally, multiple other RRT-related factors may exacerbate renal insult [64, 68]. These include rapid plasma osmolality shifts [69], dialysate temperature if higher than patient’s core temperature [70], dialyzer bio-incompatibility [71], unintended clearance of beneficial substances such as vasoactive drugs [72], and reduction in myocardial blood flow with transient myocardial stunning [73–75]. 

## Monitoring techniques

### Challenges in monitoring renal perfusion

At the macrocirculatory level, estimating RPP - particularly if derived using CVP - may be inaccurate, as it may not account for elevated IAP or the presence of intracapsular or renal interstitial hypertension. These conditions can contribute to renal congestion and reduce effective RPP, particularly in patients receiving mechanical ventilation or those with abdominal pathology or with significant fluid overload. At the microcirculatory level, monitoring renal perfusion is complicated by the kidney’s heterogeneous vasculature. Presence of several different vascular beds in the glomerular, juxtaglomerular, and medullary (inner and outer) circulation, each with associated independent regulatory mechanisms, results in significant variability in perfusion response [7, 76]. Such heterogeneity makes it difficult to extrapolate findings from the microcirculation of one region to the entire kidney. Moreover, capillary density within the kidneys varies from individual to individual and can dynamically change during the course of an illness. A decrease in capillary density either due to disease progression or inflammation can elevate intrarenal vascular resistance, altering microcirculatory hemodynamics and complicating an accurate assessment of renal perfusion [76]. 

Multi-modal monitoring of renal perfusion may be more effective in early detection of renal malperfusion among critically ill patients. As reviewed below, other monitoring tools that have recently emerged are renal biomarkers, renal Doppler ultrasound, contrast-enhanced renal ultrasound, microcirculation monitoring, tissue oxygenation monitoring, and assessment of intra-renal pressure. These techniques may offer insights into adequacy of renal perfusion in a more dynamic manner. However, most of these tools are still under evaluation and are yet to be widely adopted in clinical practice.

### Novel renal biomarkers

Biomarkers can potentially help improve early detection of renal stress or sub-clinical AKI beyond traditional markers such as serum creatinine or urine output, however no biomarker alone currently provides a direct assessment of renal perfusion. Circulating renin levels, with a short half-life of ~ 10 min and a prompt trigger response to hypotension, is well suited for dynamic monitoring and has emerged as a promising marker of renal perfusion among critically ill patients [77]. Multiple studies show that renin has strong associations with both mortality [78, 79] and new adverse kidney events [80, 81]even outperforming lactate in predicting mortality in hypotensive ICU patients [82, 83]. In addition, high renin levels may be able to identify patients more likely to benefit from exogenous angiotensin II [84], underscoring its potential as a tool to guide therapy. Work is ongoing on developing a point-of-care renin assay, which may accelerate its adoption in clinical settings.

Several other biomarkers have emerged as sensitive indicators of renal tubular stress from suboptimal perfusion. One of the most extensively studied biomarkers of kidney damage is neutrophil gelatinase-associated lipocalin (NGAL), which is a member of the lipocalin superfamily of proteins and is upregulated in renal tubular and inflammatory cells in response to injury. It has emerged as an early, sensitive, and noninvasive urinary biomarker of both ischemic and nephrotoxic AKI [85]. NGAL, measured either in serum or urine, can potentially predict AKI 24–48 h before the diagnosis of creatinine-based AKI and to detect subclinical AKI (i.e., NGAL-positive but creatinine-negative), which is associated with subsequent need for RRT or death [86–89]. Perhaps two of the most validated biomarkers are urinary tissue-inhibitor of metalloproteinases-2 (TIMP-2) and insulin-like growth factor-binding protein 7 (IGFBP7), which are released by tubular cells in response to ischemia or injury, and their product [TIMP-2] x [IGFBP7] has been shown to be an accurate predictor of all-cause AKI [90, 91]. Importantly, biomarker ([TIMP-2] x [IGFBP7]) triggered management, focused on optimizing volume status, maintaining perfusion pressure, and discontinuing nephrotoxic agents, has shown clinical benefit in high risk post-surgical ICU patients in randomized trials [92, 93]. Another biomarker, kidney injury molecule-1 (KIM-1), an immunoglobulin superfamily cell-surface protein, is not normally detectable in health, but is dramatically upregulated in an ischemic kidney in the surviving proximal epithelial cells [94, 95]. Overall, while no biomarker alone currently provides a direct assessment of renal perfusion, combining these biomarkers with clinical risk scores seems promising for early detection of renal malperfusion or risk stratification of AKI and treatment [96–98]. 

### Renal ultrasound

Intrarenal Doppler ultrasound is gaining attention for evaluating renal perfusion and congestion in critically ill patients. One of the most widely used indicators is renal resistive index (RRI), which is obtained via Doppler ultrasound of the intrarenal arteries and calculated as the difference between the peak systolic velocity (*V*_*systole*_) and end-diastolic velocity (*V*_*diastole*_) divided by the peak systolic velocity (i.e., RRI = [*V*_*systole*_– *V*_*diastole*_] / *V*_*systole*_). RRI is primarily influenced by pulse pressure and renal capillary wedge pressure, and provides a hemodynamic window to renal perfusion [99, 100]. It is a potentially reliable predictor of overall survival [101]. However, the relationship between RRI and renal vascular resistance is complex and influenced by a variety of factors [102], with one multicentre study of 371 ICU patients demonstrating that RRI, though statistically associated with development of AKI, performed relatively poorly in the prediction of persistent AKI or need for RRT [103]. As such, additional studies are needed to better delineate and validate the role of RRI in the evaluation of patients at risk of AKI in the ICU.

On the venous side, intrarenal vein Doppler can be used to assess intrarenal venous flow (IRVF) patterns. In the setting of renal venous congestion, the normal pattern of continuous IRVF is replaced by a discontinuous flow, an effect which can be quantified using the renal venous stasis index (RVSI). Discontinuous IRVF patterns or elevated RVSI are indicators of congestion or reduced intrarenal compliance that may be useful in limiting fluid resuscitation to avoid renal parenchymal congestion [104, 105]. The combination of intrarenal venous Doppler with hepatic vein and portal vein Doppler has been incorporated into a generalized assessment of intraabdominal organ congestion called the venous excess ultrasound (VExUS) score, which has gained significant traction as a method to guide fluid management in patients with heart failure or general critical illness [106–109]. 

Another monitoring technique is contrast-enhanced ultrasound (CEUS), which, in contrast to Doppler ultrasound, can be used to evaluate the microcirculation and has been used to assess tissue perfusion for several organs. CEUS quantitatively assesses alterations in renal microcirculation in real time by using highly echogenic inert microbubbles of similar size as red blood cells to map areas of perfusion [110]. The quantitative parameters obtained through CEUS are strongly correlated with RBF, which may facilitate real-time monitoring of renal microcirculatory perfusion, enabling individualized hemodynamic therapy [111]. However, data thus far on the use of CEUS in the ICU, though showing some promise in predicting the prognosis of AKI, are limited to small single-centre studies [112–114]. In addition, CEUS is complex, with a variety of factors and artefacts able to influence CEUS parameters, with some studies suggesting significant measurement variability and, for some parameters, poor reproducibility [112, 114]. Additional data are needed to better define and validate the utility of CEUS in the ICU.

### Tissue oxygenation and microcirculation monitoring

Near-infrared spectroscopy (NIRS) is a potentially useful modality to measure renal tissue oxygen saturation (rSO_2_) in real-time by detecting the regional balance between oxygenated and deoxygenated hemoglobin within a local tissue area [115, 116]. This allows dynamic monitoring of rSO_2_ over time and such trajectory analysis can better capture the risk of AKI compared to static thresholds [117]. Another promising tool that was first developed decades ago but has recently re-emerged for monitoring renal medullary oxygenation is continuous measurement of bladder urinary oxygen tension (PuO_2_), which may be considered as a window into renal medullary health [110, 118, 119], but data to support its use remain limited to animal studies and small single-centre human studies [120–122]. Currently, microcirculation monitoring is an intense focus of research but validated bed-side tools are lacking [76]. 

### Dynamic monitoring of renal autoregulation

Pressure-flow relationship in renal vasculature can be assessed dynamically by tracking changes in the measures of renal perfusion corresponding to spontaneous or induced changes in RPP [123]. As stated earlier, the autoregulatory mechanisms may be impaired or shifted in critical illness, particularly in presence of pre-existing comorbidities, making standard MAP targets less reliable to avoid malperfusion [124]. Dynamic monitoring of renal autoregulation threshold may hold some advantages in guiding towards an optimal target MAP rather than the current practice using empirically chosen targets. Optimizing renal perfusion may benefit other organs too. One study showed that the magnitude-duration of MAP below the lower autoregulation limit of cerebral blood flow was independently associated with the risk of new AKI [125]. This suggests a similar concept where the duration and degree to which RPP remains under the lower limit of renal autoregulation threshold may also be associated with AKI. Despite these promising insights, real-time assessment of renal autoregulation remains largely experimental and standardized clinical tools for bedside use are still lacking [123]. 

### Monitoring for intra-abdominal hypertension

Emerging techniques for non-invasive or minimally invasive continuous IAP monitoring are under investigation. A novel bladder pressure transduction system connected to a fluid-filled Foley catheter has shown promising results in clinical validation studies, demonstrating accurate and continuous IAP measurement without need for repeated manual readings [126]. Such technologies may allow earlier recognition and management of intra-abdominal hypertension in at-risk ICU patients.

## Therapeutic considerations

**Fig. 4:**
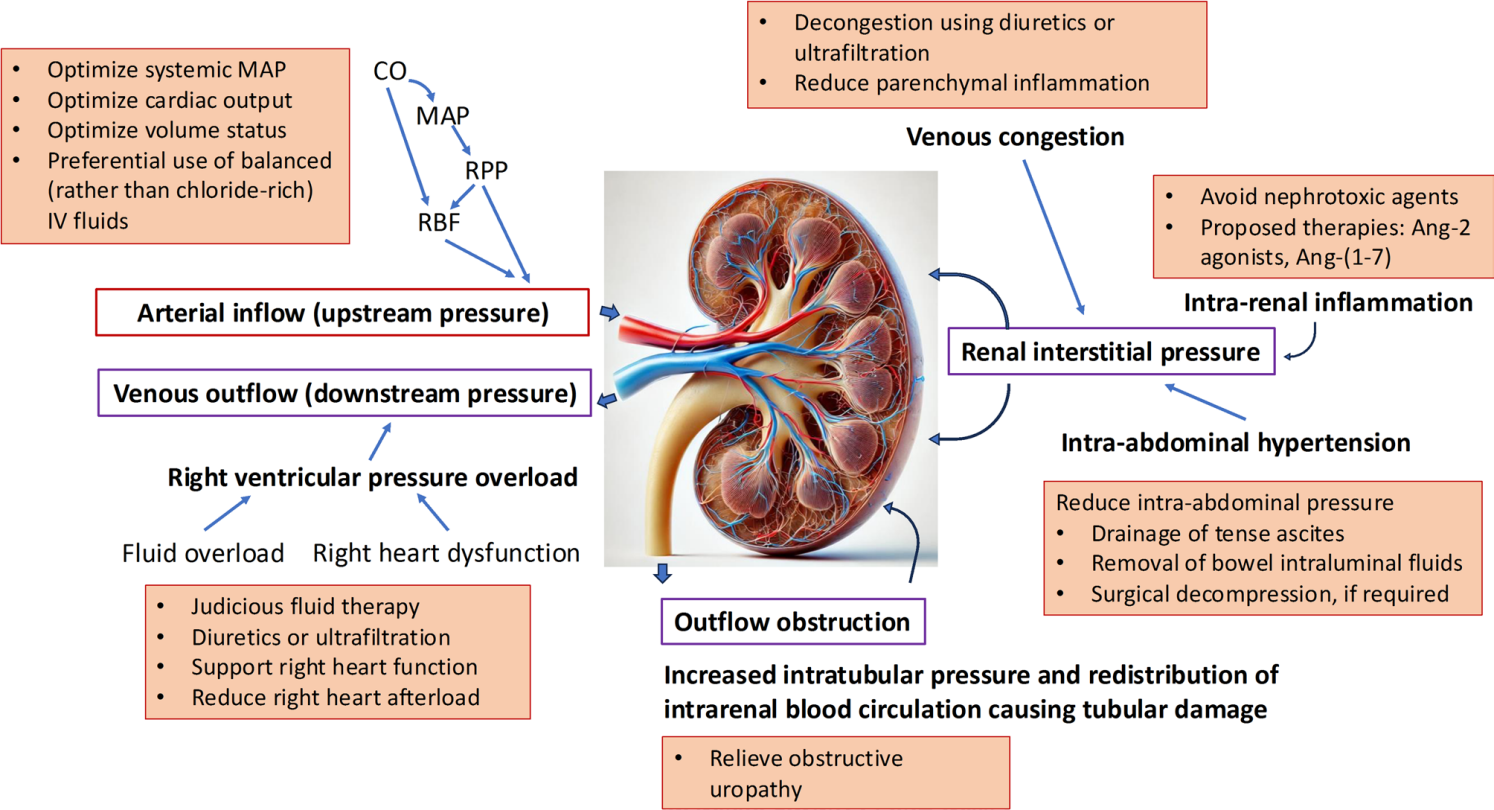
Therapeutic approaches for optimizing renal hemodynamics in critical illness Note: This diagram outlines key modifiable factors influencing renal perfusion and therapeutic strategies targeting upstream (arterial), downstream (venous), and intrarenal pressures, alongside interventions addressing systemic, cardiac, and intra-abdominal contributors to help optimize renal hemodynamics Ang-(1–7): angiotensin-(1–7); Ang-2: angiotensin II; CO: cardiac output; MAP: mean arterial blood pressure; RPP: renal perfusion pressure; RBF: renal blood flow

The key therapeutic focus in preserving renal function or facilitating renal recovery is the maintenance of macrovascular renal perfusion through attention to components of RPP - renal arterial blood pressure (upstream pressure), renal venous pressure (downstream pressure), intrarenal pressure (precipitated by tubular leakage; tubular obstruction; intra-renal inflammation, e.g., due to sepsis and/ or nephrotoxins; and intra-abdominal hypertension), and their dynamic interplay (Fig. 4). As we discuss later, another potential therapeutic target under investigation is the complex cascade of microvascular dysfunction and cellular injury impacting aspects on molecular and microanatomical level - impaired microcirculation, inflammation, immune dysregulation, and oxidative injury.

### Renal arterial blood pressure

Effective renal perfusion depends on maintaining sufficient forward flow and circulating volume. In the early stages of shock, timely restoration of cardiac output through judicious fluid resuscitation and prompt vasopressor initiation is essential. Observational studies suggest that lower MAP targets are associated with poorer renal outcomes in critically ill patients [127]. However, results from previous RCTs [128–131] evaluating different blood pressure targets in critically illness or perioperative settings have been inconsistent. This may be explained by varied methodologies, patient populations, geographic settings, and the limitations of a one-size-fits-all approach associated with utilizing uniform MAP targets [132]. There is growing recognition that relative hypotension may represent an important target for hemodynamic optimization [133, 134]. However, aside from a few small pilot trials [135, 136] and an RCT conducted in non-ICU population [137], high-quality evidence is lacking to guide optimal strategies for individualizing RPP in ICU patients with shock. RPP or mean perfusion pressure (MPP), as a close surrogate, is increasingly regarded as a reliable marker compared to MAP or CVP alone for predicting risk of new-onset AKI [138–140]. In critically ill patients, reduced MPP, larger MPP deficits, and more time spent with a MPP deficit > 20% relative to baseline have been linked to a higher risk of developing AKI [139–143]. In contrast, a retrospective cohort study in cardiac surgery patients reported no association between MPP deficit and AKI progression [144]. Differences in patient populations, vasopressor exposure, MPP assessment periods, confounders considered in multivariable analyses, definitions for AKI onset, and time windows for AKI progression, including underlying pathophysiology, could account for this discrepancy. Increasingly, focus has shifted toward evaluating individualized MAP targets tailored to patients’ pre-illness blood pressure, as such an approach may be associated with improved outcomes [135, 141–143]. While promising, this concept remains unexplored in large trials, though a multicenter RCT is currently underway (NCT05850962).

### Fluid management while avoiding overload

Effective fluid management plays a pivotal role in renal perfusion. Elevated CVP, a surrogate for renal venous pressure, has been independently associated with increased AKI risk, with lower CVP levels (below 8 mmHg) correlating with better renal outcomes [139, 145]. Liberal fluid administration may worsen AKI risk by causing renal congestion and interstitial edema and impairing microvascular oxygen delivery [146–148]. Importantly, however, thus far RCTs evaluating protocolized fluid restriction in critically ill patients have had largely mixed outcomes. These trials suggest that fluid restriction is safe although without any mortality benefit in septic shock. Such strategies may reduce the duration of mechanical ventilation in patients with acute respiratory distress syndrome, however may increase the risk of AKI in major abdominal surgery [149–152]. Importantly, while most experts agree that early aggressive fluid resuscitation remains appropriate for patients with septic shock, subsequent caution with fluids is prudent to mitigate harms associated with volume overload [51, 153]. However, the optimal timing of transition between these two approaches remains to be precisely defined. Additional multicentre trials of fluid restriction in sepsis are ongoing (NCT04569942, NCT05179499).

The composition of fluids may also matter, as chloride-rich solutions can impair renal perfusion through vasoconstriction and altered TGF [154, 155]. Though large RCTs [156–158] have yielded somewhat mixed results and the effect size appears small, a recent Bayesian meta-analysis [159] concluded that there is a high probability that use of balanced crystalloids in critical illness reduces mortality.

### Vasoactive agents

Following initial volume resuscitation and restoration of adequate volume state, further improvement in renal perfusion in shock states generally requires vasopressor support [160]. Comparative RCTs [161–164] have shown that while overall clinical outcomes may be similar, vasopressin instead of or in addition to norepinephrine seems to be associated with better renal function preservation than use of norepinephrine alone in select high-risk patients.

The potential renal benefit of vasopressin may stem from its minimal vasoconstrictive effect on renal afferent arterioles and preferential vasoconstriction of efferent arterioles, thus maintaining glomerular pressure and GFR in the setting of compromised renal perfusion. As outlined in further detail below, the newer vasoconstrictor angiotensin II also preferentially acts on the efferent arteriole and therefore may also be useful to maintain kidney function in patients with shock, though thus far human studies to support this possible benefit are limited to post hoc analyses of trial data [165, 166]. Though typically attributed to their differential effects on afferent vs. efferent arteriolar vasoconstriction, animal studies suggest that the renal benefits of vasopressin and angiotensin II could be mediated by differential effects on renal medullary perfusion and oxygenation [167, 168]. 

Specifically, the single-centre VANCS trial suggested a beneficial effect of vasopressin on the incidence of AKI in patients with vasoplegia after cardiac surgery [164], but this finding has yet to be validated in multicentre trials. In septic shock, secondary renal outcomes favouring vasopressin were observed in both the VASST and VANISH RCTs [162, 163], but the primary outcomes of each study, namely mortality and days free of stage 3 AKI, respectively, were neutral. A subsequent individual patient data meta-analysis showed that vasopressin was associated with reduced need for renal RRT [169], but the finding was not robust to sensitivity analyses and the result was primarily driven by the VANISH trial [164], in which RRT use was reduced in non-survivors but not in survivors. Additionally, observational data suggest that outcomes with vasopressin may vary based on timing and patient selection, with worse outcomes observed at higher doses and among patients with elevated lactate [170]. 

Likewise, the renal benefits of vasopressin and angiotensin II have been suggested by a recent meta-analysis evaluating the effects of non-adrenergic vasopressors in septic shock and post-operative vasoplegia. This meta-analysis found that, in an effect largely driven by vasopressin and angiotensin II, non-adrenergic agents were associated with decreased need for RRT [171]. Interestingly, several RCTs on vasopressin and angiotensin II predate the publication of the recent series of RCTs on the timing of initiation of RRT which collectively have shown no benefit to routine early use of RRT [65, 172, 173]. Therefore, future trials in the setting of modern RRT prescribing practices are required to validate such a benefit. Importantly, RCTs of systemic renal vasodilators like fenoldopam, natriuretic peptides, or levosimendan have not shown clinical benefit in terms of improving RBF, and have failed to reduce AKI incidence or mortality, despite plausible physiological mechanisms [174–176]. 

### Reducing renal interstitial pressure

Another important target for optimizing RPP is managing renal interstitial pressure. Beyond judicious fluid therapy, a key strategy is active decongestion using diuretics or ultrafiltration. Additional measures include optimizing right heart function (e.g., by reducing pulmonary hypertension) and alleviating pericardial, intrathoracic, or intra-abdominal pressures through drainage when necessary. Blood purification techniques using adsorption filters aimed at improving renal perfusion by reducing systemic inflammation are under investigation. In one single-center retrospective study, use of the oXiris^®^ hemofilter was associated with improved renal perfusion parameters based on ultrasound imaging, although clinical outcomes were no different in the treated and untreated groups and conclusive evidence to support this or other blood purification techniques remains lacking [177]. Early and targeted management of the underlying cause of AKI may also reduce intra-renal inflammation and support recovery. Elevated IAP can exacerbate renal interstitial pressure and impair RPP. Prompt recognition of intra-abdominal hypertension and timely interventions, such as fluid removal, bowel decompression, abdominal drainage, or surgical decompression, may improve renal perfusion and clinical outcomes [57]. Similarly, relieving urinary tract obstruction is critical for reducing intratubular pressure, restoring RBF distribution, and preventing acute tubular injury.

### Countering inflammatory mediators

Inflammatory pathways, particularly in contexts of gram-negative sepsis, ischemia-reperfusion injury, or malignancy, can play a major role in the progression of AKI. These insults can trigger the release of pro-inflammatory cytokines and chemokines, which in turn activate dysregulated immune cell populations including neutrophils, macrophages, and natural killer cells [178]. While these mechanisms are well described in preclinical models, robust human evidence remains limited. In the STOP-AKI and REVIVAL RCTs, despite secondary outcomes suggesting benefit, treatment with human recombinant alkaline phosphatase failed to significantly improve the primary trial outcomes of kidney function and 28-day mortality, respectively, in critically ill patients with sepsis-associated AKI [179, 180]. Extracorporeal blood purification techniques aimed at removing circulating inflammatory mediators remain an area of active investigation, but definitive clinical benefit has yet to be demonstrated.

### Renal replacement therapy

In view of its potential contribution to renal injury, recent studies have focused on defining safer practices in RRT prescription. Comparative RCTs of intermittent versus continuous RRT modalities have yielded inconsistent results in terms of survival or kidney recovery [181, 182]. Regarding continuous RRT, an individual patient data meta-analysis suggests that higher intensity RRT (prescribed dose higher than 20–25 mL/kg/h) can delay renal recovery, and should therefore be avoided in most patients [183]. Additionally, there is some evidence suggesting that higher net ultrafiltration rate (exceeding 1.75 mL/kg/h) may also delay renal recovery and perhaps should be used with caution [184]with the possible exception of cases of overt severe volume overload [185]. These concerns maybe particularly relevant in critically ill patients with acute-on-chronic kidney disease [186]. 

### Antioxidants

Oxidative stress, largely mediated by free oxygen radicals, may also contribute to renal injury under similar pathological conditions, including contrast exposure and chemotherapy. Antioxidant agents such as alpha-lipoic acid, selenium, sodium-2-mercaptoethane sulphonate (MESNA), and curcumin are some potential therapies that directly target free radicals. Despite promising animal studies, human data remains ambiguous or lacking. In the SUSTAIN CSX RCT, high-dose intravenous sodium selenite demonstrated no improvement in postoperative organ dysfunction or mortality in high-risk cardiac surgery patients [187]. Likewise, on the basis of observational studies, the antioxidant vitamin C was felt to have potential as a treatment in sepsis, but subsequent multicenter trials have shown no benefit or signals for harm [188–190]. 

### Role of intravenous amino acids

Intravenous amino acid (L-Alanyl-Glutamine, L-amino acids, L-glutamic acids, peri-operative Custodiol^®^ solution) have been investigated for their potential reno-protective effects through recruitment of renal functional reserve. Proposed mechanisms of action include increased renal perfusion, improved renal oxygenation, and increased GFR, which are likely achieved via decreased afferent arteriolar resistance, increased renal unit plasma flow, decreased TGF, and upregulated cortical nitric oxide synthase activity [191–194]. The PROTECTION RCT found that infusion of a balanced amino acid mixture reduced AKI incidence in patients undergoing cardiac surgery [195]. However, it remains uncertain whether this benefit reflects true renal protection at the tubular level, or only a functional improvement in perfusion, or both [196]. Two recent systematic reviews and meta-analyses, each including over 4500 patients surgical patients (although heavily weighted by the PROTECTION RCT), suggested that intravenous amino acids reduced the incidence of postoperative AKI and increased urine output, in patients at high risk of AKI, particularly after cardiac and major vascular surgery, but neither found a beneficial effect on the rate of RRT use or mortality [197, 198]. Moreover, the only RCT in critically ill ICU patients showed no significant benefit even with regards with AKI [199]. 

### Restoring microvascular circulation

Beyond macrovascular flow disturbances, perturbations in renal microcirculation can also play a critical role in the development of AKI [200]. Emerging therapies are exploring ways to mitigate the downstream effects of microvascular dysfunction and cellular injury in AKI. Targets include impaired microcirculation (e.g., angiotensin II, adenosine receptor antagonists), inflammation (e.g., alkaline phosphatase, sphingosine-1-phosphate analogues, dipeptidyl peptidase-3 inhibitors), immune dysregulation, oxidative stress (e.g., antioxidants such as alpha-lipoic acid, curcumin, Na-2-MCE, propofol, selenium), and transcriptional pathways modulated by genetic regulators such as 5INP [33, 201, 202]. 

Novel agents such as angiotensin II and adenosine receptor antagonists are being investigated for their ability to modulate microvascular tone and restore renal perfusion. Angiotensin II regulates the release of aldosterone and vasopressin and, through both these mediators and direct effects on the kidney, plays a key role in maintaining sodium and water homeostasis [203, 204]. During sepsis and inflammation, downregulation of angiotensin I type of angiotensin receptors and relative deficiency of angiotensin II have been observed, which may compromise renal perfusion [205]. Exogenous angiotensin II has been shown to maintain tissue perfusion and organ function in both animal and human studies of septic shock, although additional trials are needed to confirm its clinical utility [206, 207]. Another experimental therapy is Angiotensin 1–7, a Mas receptor agonist, and a major effector molecule of the alternative renin-angiotensin system which can modulate oxidative stress, inflammation, and endothelial injury [208]. In animal models of renal ischemia, activation of the alternative renin-angiotensin system, via administration of exogenous angiotensin 1–7 or an angiotensin II type 2 receptor agonist, mitigated oxidative stress and renal inflammation, alleviating ischemia-reperfusion induced kidney injury [209, 210]. 

Adenosine is another key mediator of renal microvascular tone. In response to hypoxia or increased tubular sodium chloride delivery, adenosine induces afferent arteriole vasoconstriction, thereby reducing GFR [211]. This mechanism can be inhibited by adenosine receptor antagonists, including nonselective agents like theophylline and aminophylline, and selective A1 receptor blockers such as rolofylline. However, clinical studies so far have yielded limited clinical benefit and conflicting results [212–214]. 

Taken together, although these microvascular interventions hold physiological promise, their clinical utility remains uncertain. As stated above, evidence from animal models and human observational studies suggests that early fluid resuscitation and appropriate vasopressor therapy may help mitigate sepsis-induced microcirculatory dysfunction and may beneficially modulate the microcirculation [25, 47–49]. These findings also highlight that microcirculatory flow is influenced by macro-hemodynamic variables such as elevated CVP and reduced MAP, indicating a dynamic interplay between the macrocirculation and microcirculation [25, 47–49, 215, 216]. Thus, the notion that AKI in sepsis is driven solely by either microcirculatory or macrocirculatory dysfunction is likely a false dichotomy. Nonetheless, in the absence of clinically validated tools to assess or treat microcirculatory dysfunction at the bedside, macro-circulatory optimization remains central to the prevention and management of AKI in critically ill patients [92, 93, 217–219]. 

## Future directions

Despite substantial progress in understanding of renal hemodynamics, there are significant knowledge gaps and research opportunities. RPP or MPP (as a close surrogate) is increasingly regarded as a reliable marker compared to MAP or CVP alone. These macrocirculatory surrogate markers, however, may not correlate well with renal microcirculatory perfusion, which is a function of several other factors. Future research must address key gaps in our understanding of microvascular dynamics and renal autoregulation capacity. Rather than static or uniform MAP targets often pursued in real-world practice, there is a pressing need to shift toward dynamic RPP-guided hemodynamic strategies that can integrate real-time tissue perfusion monitoring to allow more individualized hemodynamic adjustments. Multimodal monitoring - using intrarenal Doppler imaging, contrast-enhanced ultrasound, urinary bladder oxygen sensors, microcirculatory imaging tools, tissue oxygenation monitoring, assessment of intra-renal pressure, and plasma and urinary biomarkers - can provide a real-time overview of adequacy of renal tissue perfusion. When integrated at the bedside, these technologies may detect hypoperfusion earlier, guide individualized RPP targets, and enable precision interventions tailored to patient-specific pathophysiology. However, though promising, the use of these novel tools is currently supported by only preliminary data and additional multicentre studies are required to better define and validate their use. In addition, future research should focus on defining personalized RPP thresholds, as optimal perfusion likely varies with comorbidities, baseline renal function, and the nature of critical illness. Machine learning models trained on multi-parametric data could assist in risk stratification and early warning of renal malperfusion. Additionally, much of the current literature focuses on short-term outcomes such as in-hospital AKI and mortality. However, the long-term consequences of sustained RPP derangements, including progression to chronic kidney disease and long-term dialysis dependence, remain underexplored. Prospective cohort studies with extended follow-up are essential to understanding the broader impact of RPP management during critical illness.

## Data Availability

Not applicable.
